# Reduced mortality and subsequent fracture risk associated with oral bisphosphonate recommendation in a fracture liaison service setting: A prospective cohort study

**DOI:** 10.1371/journal.pone.0198006

**Published:** 2018-06-01

**Authors:** Tineke A. C. M. van Geel, Dana Bliuc, Piet P. M. Geusens, Jacqueline R. Center, Geert-Jan Dinant, Thach Tran, Joop P. W. van den Bergh, Alastair R. McLellan, John A. Eisman

**Affiliations:** 1 Department of Family Medicine, Maastricht University, Research School CAPHRI, Maastricht, The Netherlands; 2 Osteoporosis & Bone Biology Program, Garvan Institute of Medical Research, Sydney, Australia; 3 Department of Internal Medicine, Subdivision of Rheumatology, Maastricht University Medical Center, Research School CAPHRI, Maastricht, The Netherlands; 4 Biomedical Research Institute, University Hasselt, Hasselt, Belgium; 5 Faculty of Medicine, University of New South Wales (UNSW) Sydney, Australia; 6 Clinical School, St Vincent's Hospital, Sydney, Australia; 7 Department of Internal Medicine, Subdivision of Rheumatology, Maastricht University Medical Center, Research school NUTRIM, Maastricht, The Netherlands; 8 Department of Internal Medicine, VieCuri Medical Centre of Noord-Limburg, Venlo, The Netherlands; 9 Western Infirmary, Gardiner Institute, Glasgow, United Kingdom; 10 Clinical Translation and Advanced Education, Garvan Institute of Medical Research, Sydney, Australia; 11 School of Medicine, University of Notre Dame Australia, Sydney, Australia; University of Nevada Las Vegas, UNITED STATES

## Abstract

**Objective:**

Osteoporotic fragility fractures, that are common in men and women, signal increased risk of future fractures and of premature mortality. Less than one-third of postmenopausal women and fewer men are prescribed active treatments to reduce fracture risk. Therefore, in this study the association of oral bisphosphonate recommendation with subsequent fracture and mortality over eight years in a fracture liaison service setting was analysed.

**Materials and methods:**

In this prospective cohort study, 5011 men and women aged >50 years, who sustained a clinical fracture, accepted the invitation to attend the fracture liaison service of the West Glasgow health service between 1999 and 2007. These patients were fully assessed and all were recommended calcium and vitamin D. Based on pre-defined fracture risk criteria, 2534 (50.7%) patients were additionally also recommended oral bisphosphonates. Mortality and subsequent fracture risk were the pre-defined outcomes analysed using Cox proportional hazard models.

**Results:**

Those recommended bisphosphonates were more often female (82.9 vs. 72.4%), were older (73.4 vs. 64.4 years), had lower bone mineral density T-score (-3.1 vs. -1.5) and more had sustained hip fractures (21.7 vs. 6.2%; p < 0.001). After adjustments, patients recommended bisphosphonates had lower subsequent fracture risk (Hazard Ratio (HR): 0.60; 95% confidence interval (CI): 0.49–0.73) and lower mortality risk (HR: 0.79, 95%CI: 0.64–0.97).

**Conclusion:**

Of the patients, who are fully assessed after a fracture at the fracture liaison service, those with higher fracture risk and a recommendation for bisphosphonates had worse baseline characteristics. However, after adjusting for these differences, those recommended bisphosphonate treatment had a substantially lower risk for subsequent fragility fracture and lower risk for mortality. These community-based data indicate the adverse public health outcomes and mortality impacts of the current low treatment levels post fracture could be improved by bisphosphonate recommendation for both subsequent fracture and mortality.

## Introduction

Osteoporotic fragility fractures are common in men as well as women. More than 50% of women and more than 25% of men aged older than 50 years will sustain a fragility fracture in their remaining lifetime.[1–4] Moreover, it is clear that initial fragility fractures signal substantially increased risk of further fractures.[5, 6] Several data also support the relationship between major (proximal) fragility fractures and premature mortality.[7–21] Secondary fracture prevention, using pharmacological treatments for osteoporosis, happens relatively rarely for women and even more rarely for men. Although it is validated in randomised controlled trials (RCTs) and endorsed by all national guidelines.[5, 22, 23] Worldwide, there is a rising call for implementing fracture liaison services for secondary fracture prevention.[5, 22, 23] A recent review [24] reported improvement of treatment of postmenopausal US women after a fracture, but this still occurred in less than 30%. Men do worse after such fractures in terms of future fracture risk and in terms of excess mortality.[6–10, 16, 20] In some jurisdictions, the lack of implementation may relate to concerns about the robustness of evidence for the clinical and societal benefit in the “real world”. Recent studies of osteoporosis pharmacotherapy have reported statistically and clinically significant survival benefits in one RCT,[25] a meta-analysis of prior RCTs,[26] and population-based studies.[27, 28] It is suggested that RCTs of fracture liaison services after prior fractures are required to evaluate fracture risk reduction and survival effects in the general community. However, such trials are unlikely given the major ethical challenges of randomising some participants to less than recommended care.[29]

For that reason, the risk of subsequent fractures and mortality over an 8-year follow-up period was evaluated in patients with fractures attending the West Glasgow Fracture Liaison Service in relation to recommendations for osteoporosis specific therapy.

## Materials and methods

### Study design and participants

Between 1999 and 2007, patients aged 50 years and over with a low trauma fracture at accident & emergency/trauma and orthopaedic fracture services were identified by osteoporosis nurse specialists of the Fracture Liaison Service of the West Glasgow health service, as previously described.[30, 31] Low trauma was defined as no obvious cause or minimal trauma such as a fall from standing height or less.

Patients were assessed approximately six weeks post fracture. Treatment was typically started around two weeks later by the patient's general practitioner (GP). [30, 31] Treatment was recommended for five years in first instance and GPs were advised to arrange DXA monitoring after that interval. [30] Eight years later, patients were followed up to see whether they had sustained any subsequent fractures and whether they were still alive. All fractures were radiographically confirmed. Deaths were confirmed by hospital records, which were updated quarterly from the records of the office of the Scottish registrar of deaths. Specific adherence data are not available.

### Exposure

For those attending the Fracture Liaison Service, a treatment recommendation based on pre-defined criteria related to assessment of future potential fracture risk and endorsed by the lead consultant was provided to the patient’s general practitioner. All patient data with regard to fracture and medical history, risk factors for osteoporosis and fractures, lifestyle, (and if indicated) osteoporosis treatment recommendations, and arrangements for follow-up were stored in a computerised database.[30, 31] Blood samples were collected to exclude secondary osteoporosis.[32] All patients who had such potential causes identified were recommended appropriate treatments as per national guidelines.

All attendees were recommended oral 1000 mg calcium, as carbonate, and 800 IU vitamin D daily reflecting the regimen by Chapuy et al.[33] Patients, meeting pre-specified criteria also had oral bisphosphonate therapy recommended.[30] Oral bisphosphonate therapy was identified as the treatment of choice and recommended in accordance with evidence-based practice and national osteoporosis guidelines at that time, unless there was a clear contraindication.[30] It was recommended based on the following predefined criteria: site (and number) of fractures and lowest T-score at femoral neck, total hip or lumbar spine according to the patient’s age.

Patients aged 50 years or older were recommended oral bisphosphonate if they had had:

- two or more vertebral fractures irrespective of patient’s age or bone mineral density (BMD) T-score, or
- only one prior vertebral fracture and BMD ≤ -2.0 (age 50–59 years) or ≤ -1.6 (aged ≥ 60 years), or
- any other prior non-vertebral fracture & BMD ≤ -2.5 (age 50–59 years) or ≤ -2.0 (≥ 60 years).

Note that in order to receive a bisphosphonate recommendation, patients did not need to have an osteoporotic T-score (≤ -2.5).

### Main outcome measures and covariates

Mortality and subsequent fracture rates were the pre-defined outcomes. The following baseline characteristics were included in the analyses: gender, age, weight, height, body mass index (BMI), femoral neck and lumbar spine BMD T-score, initial fracture type: hip, major (pelvis, distal femur, proximal tibia, multiple rib, proximal humerus, clinical vertebra) or minor (all other). These major and minor fracture groupings were chosen as they had been previously shown to relate to mortality outcomes.[34] Also included were recognised health conditions and co-morbidities, such as: smoking status, past or current glucocorticoid use, presence of rheumatoid arthritis, inflammatory bowel disease, family history of osteoporosis, a maternal history of hip fracture, thyrotoxicosis and alcohol intake ≥ 5 units/day. Note that this criterion for alcohol excess pre-dated the FRAX^®^ categorisation and was unequivocally excessive.

### Statistical analyses

The analyses in this paper are focused on those patients, who accepted the invitation for full assessment. Therefore, the data presented are only for those subjects who attended the FLS clinic and received bisphosphonate treatment recommendations (or not) based on pre-defined criteria. The outcomes of subsequent fractures and mortality over an 8-year follow-up period was analysed using SPSS (version 21.0). Those patients recommended oral bisphosphonates (plus calcium and vitamin D) were compared with those who were recommended calcium and vitamin D alone. A small number (2.5%) of individuals recommended hormone therapy (HT), strontium ranelate or teriparatide, were excluded.

Some individuals did not receive the pre-defined recommendations; i.e. were recommended bisphosphonate when they did not meet the criteria or did not receive that recommendation when they did meet them. These individuals were assessed both according to recommendations given (primary analyses) and to the pre-defined criteria (sensitivity analyses).

### Primary analyses

Firstly, independent sample T-test and Chi-square tests were performed to compare baseline characteristics of patients who were recommended oral bisphosphonates (plus calcium and vitamin D) versus those who were recommended calcium and vitamin D alone (Table 1).

**Table 1:** Characteristics of patients prescribed oral bisphosphonates compared with those prescribed only calcium and vitamin D.

	Vitamin D and calcium alone (n = 2477)	Bisphosphonates plus vitamin D and calcium (n = 2534)	P-value
Gender, n (%)			< 0.001
Women	1793 (72.4)	2100 (82.9)	
Men	684 (27.6)	434 (17.1)	
Age, mean (SD) years	64.4 (10.2)	73.4 (9.3)	< 0.001
Weight, mean (SD), kg ^a^	75.9 (16.5)	62.8 (12.9)	< 0.001
Height, mean (SD), cm ^a^	163 (8.4)	157 (8.0)	< 0.001
Body mass Index, mean (SD), kg/m^2^ ^a^	28.7 (5.8)	25.4 (4.7)	< 0.001
Lowest T-score, mean (SD) ^b^	-1.5 (0.96)	-3.1 (0.76)	< 0.001
T-score Femoral Neck, mean (SD)	-1.2 (0.97)	-2.5 (0.80)	< 0.001
T-score Lumbar Spine, mean (SD)	-1.1 (1.21)	-2.8 (1.03)	< 0.001
Initial fracture type, n (%)^c^			< 0.001
Hip	154 (6.2)	551 (21.7)	
Major	513 (20.7)	620 (24.5)	
Minor	1810 (73.1)	1363 (53.8)	
Alcohol intake ≥ 5 units/day, n (%)	258 (10.4)	216 (8.5)	0.02
Smoking, n (%)	675 (27.3)	700 (27.6)	0.767
Past or current glucocorticoids, n (%)	47 (1.9)	71 (2.8)	0.04
Rheumatoid Arthritis, n (%)	26 (1.0)	68 (2.7)	< 0.001
Inflammatory Bowel Disease, n (%)	24 (1.0)	19 (0.7)	0.400
Family history of osteoporosis, n (%)	287 (11.6)	333 (13.1)	0.10
Maternal history of hip fracture, n (%)	173 (7.0)	181 (7.1)	0.83
Thyrotoxicosis, n (%)	30 (1.2)	68 (2.7)	< 0.001

^a^ Height, weight and body mass index (BMI) data were available in 1739 (70%) of those not on bisphosphonates treatment and 1490 (59%) of those on bisphosphonates treatment.

^b^ T-score based on lowest value of BMD at lumbar spine or femoral neck sites.

^c^ Hip, major (pelvis, distal femur, proximal tibia, multiple rib, proximal humerus, clinical vertebra) or minor (all other)

Secondly, the proportional hazard assumption checked using Schoenfeld residuals. Tests were performed to check for interaction. If interaction was present, this was added in the model. Univariable and multivariable Cox proportional hazard models were used. All variables (Table 1) were considered in the univariable analysis. Different thresholds for the P-value were used for inclusion in the multivariable analysis and for selection of the final model:

(i) In univariable models, a P ≤ 0.10 was used for selection of potentially important covariates to be included in the entry multivariable model, [35] and
(ii) In the multivariable model, the more stringent P ≤ 0.05 was used in the backward stepwise approach for selection of independent predictors in the final multivariable model.

For the final multivariable models, the backward procedure was used, excluding variables above a value of P > 0.05. All variables not stated in Tables 2 or 3, are excluded due to P-value > 0.05.

**Table 2:** Predictors of subsequent fractures. Multivariable Cox regression model; values are presented as hazard ratios (HR) with 95% confidence intervals (95%CI).

Subsequent fractures	HR (95%CI)	p-value
Gender (women)	1.63 (1.29–2.05)	<0.001
Increasing age (per 5 years)	1.06 (1.02–1.11)	0.011
Worse T-score (per 0.5 SD) ^a^	1.19 (1.14–1.25)	<0.001
Alcohol intake ≥ 5 units/day	1.98 (1.52–2.57)	<0.001
Smoking	1.30 (1.08–1.55)	0.005
**Bisphosphonates**	**0.60 (0.49–0.73)**	**<0.001**

^a^ T-score based on lowest value of lumbar spine or femoral neck

**Table 3:** Predictors of mortality. Multivariable Cox regression model; values are presented as hazard ratio’s (HR) with 95% confidence intervals (95%CI).

Mortality	HR (95%CI)	p-value
Gender (women)	0.55 (0.46–0.67)	<0.001
Increasing age (per 5 years)	1.42 (1.35–1.49)	<0.001
Worse T-score (per 0.5 SD) ^a^	1.10 (1.05–1.15)	<0.001
Initial fracture ^b^		
Hip	1.46 (1.19–1.81)	<0.001
Major	1.30 (1.07–1.58)	0.008
Minor	Reference	
Alcohol intake ≥ 5 units/day	1.70 (1.31–2.20)	<0.001
Smoking	1.82 (1.51–2.19)	<0.001
Past or current GC use ^c^	1.87 (1.23–2.85)	0.003
**Bisphosphonates**	**0.79 (0.64–0.97)**^**d**^	**0.022**

^a^ T-score based on lowest value of lumbar spine or femoral neck

^b^ Hip, major (pelvis, distal femur, proximal tibia, multiple rib, proximal humerus, clinical vertebra) or minor (all other)

^c^ GC: glucocorticosteroids

^d^ After including subsequent fractures in the model for mortality, the HR and 95%CI for bisphosphonates was essentially unchanged (HR: 0.79 (0.64–0.97)

For mortality as a main outcome measure, time was defined as time to death or was censored at eight years of follow-up. Similarly for subsequent fracture, time was defined as time to subsequent fracture or censored at eight years or death. In each analysis, gender specific analyses were performed. Treatment (oral bisphosphonates (plus calcium and vitamin D) or calcium and vitamin D alone) were analysed as co-variate.

#### Sensitivity analyses

Sensitivity analyses were performed for women and men analysed separately. Also only patients who were correctly classified according to pre-defined criteria were analysed, i.e. the other patients excluded.

### Ethics statement

This was an evaluation of the outcomes of a clinical service that had been implemented and commissioned by the National Health Service (NHS). Therefore, ethical approval was not required.

AMcL was the developer and head of this first-in-the-world fracture liaison service and was responsible for the development of the treatment criteria according to then existing guidelines. He was thus involved in patient care delivery. Data was anonymized prior to access for any analyses.

## Results

**Fig 1:**
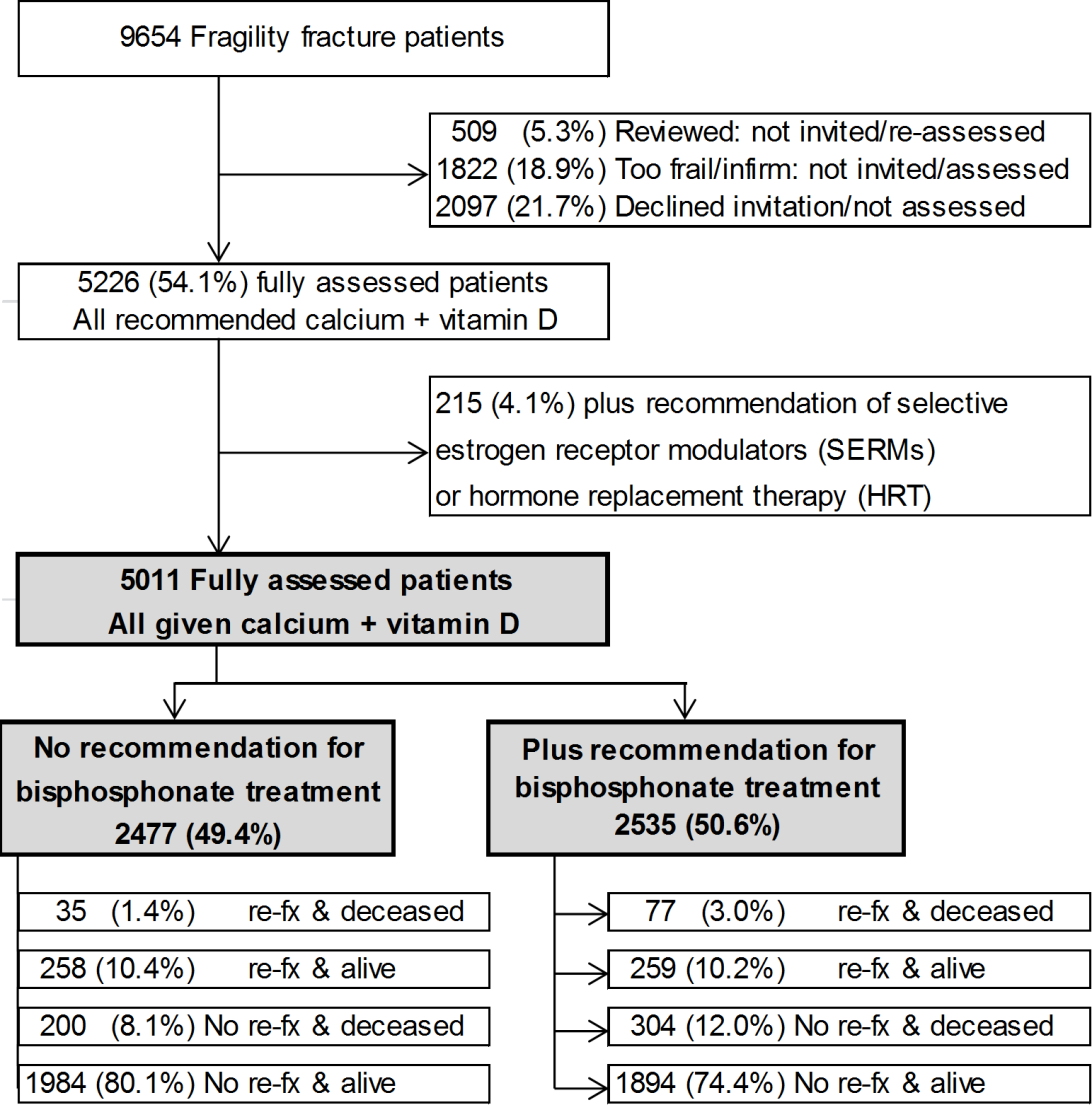
Patient disposition. All patients with low trauma fractures were invited to attend the Fracture Liaison service clinics excluding those already on treatment, considered unsuitable for treatment and/or too frail and elderly and/or declined to attend. The data and analyses herein relate to those who attended and were fully assessed. Re-fx: Subsequent fracture.

A total of 5011 (53.1%) of 9439 men and women accepted the invitation to attend the fracture liaison service and were fully assessed. The remaining 46.9% did not attend or were not assessed, either because they were already receiving treatment (5.4%), were considered by treating staff not to be candidates for further intervention beyond calcium and vitamin D (19.3%) or too frail and infirm to attend or declined to attend (22.2%; Fig 1).

### Patient characteristics

The majority of the fully assessed patients (n = 5011) were either osteoporotic (45.5%) or osteopenic (42.0%). Only 12.5% were found to have a normal BMD at all measured sites. Oral bisphosphonates were recommended in 2534 patients (50.6%) based on the pre-defined criteria of fracture type, age and lowest T-score, while 2477 patients were not recommended any additional specific treatment, apart from calcium and vitamin D (Table 1).

Those recommended oral bisphosphonates were more likely to be women (82.9 vs. 72.4%). However, as expected based on the pre-defined criteria, they were older (73.4 vs. 64.4 years), had a lower BMI (25.4 vs. 28.7 kg/m^2^) and worse BMD T-score (-3.1 vs. -1.5) and had had more ‘major’ fractures at baseline (21.7 vs 6.2% for hip, p < 0.001). They reported significantly more rheumatoid arthritis, thyrotoxicosis (p < 0.001) or to have used glucocorticoids (p = 0.035). Alcohol intake ≥ 5 units per day was a little more likely in those who did not receive a recommendation for oral bisphosphonates (10.4 vs. 8.5%, p = 0.022, Table 1).

### Subsequent fractures

**Fig 2:**
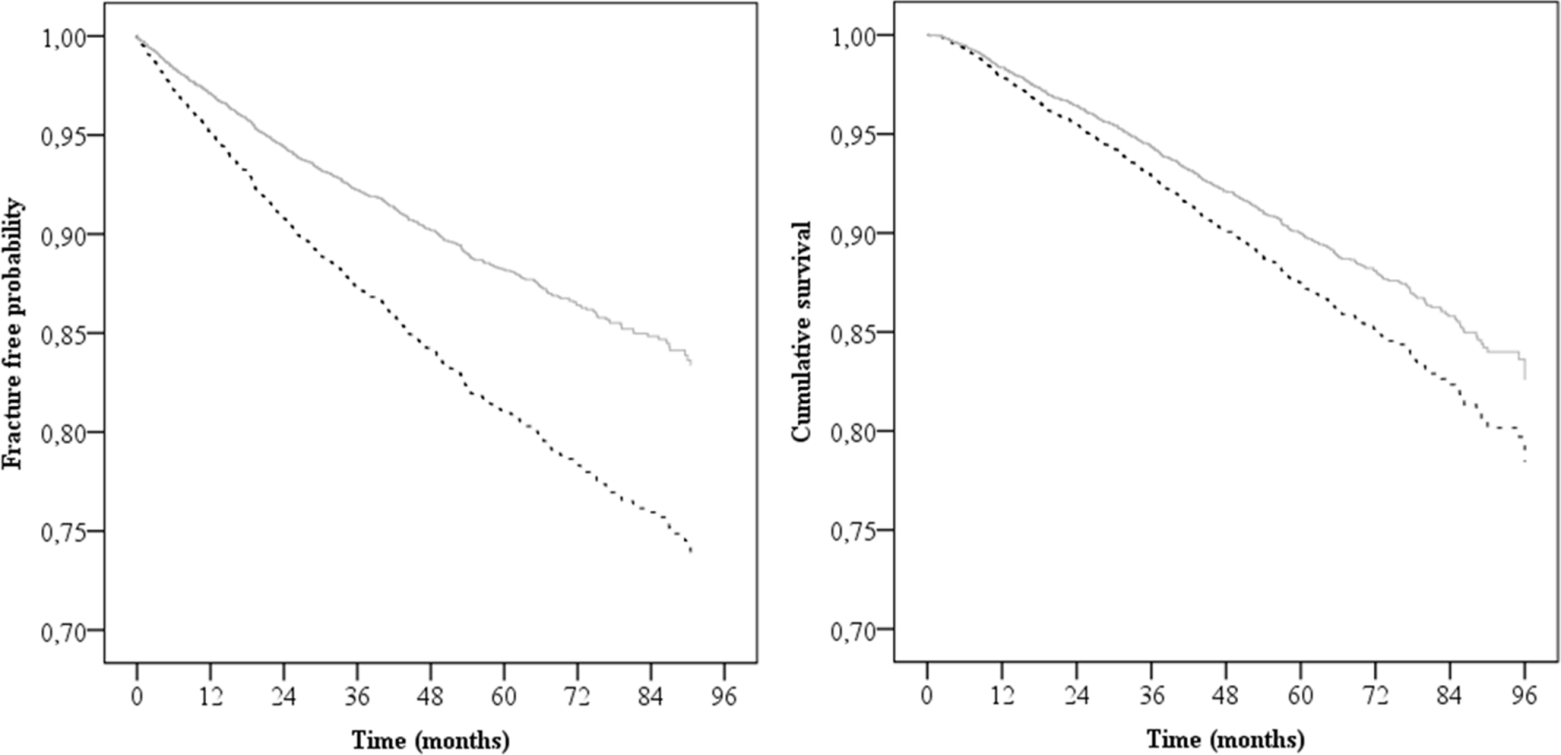
Survival curves for subsequent fractures and mortality. The curves are based on multivariable Cox proportional hazard models for subsequent fractures and mortality expressed as (A) fracture-free probability and (B) cumulative survival. Results presented for vitamin D and calcium recommendation alone (dotted black) and for bisphosphonates plus calcium and vitamin D recommendation (solid grey).

Patients who were recommended oral bisphosphonates had higher absolute subsequent fracture risk (13.3% vs. 11.8%) over a mean follow-up of 40.9 vs. 42.7 months. However, given the adverse criteria required to ‘trigger’ the recommendation for bisphosphonate use, they had a higher underlying risk (Table 2). After adjustments for these adverse criteria, they had a significantly lower subsequent fracture hazard risk (HR: 0.60, 95%CI: 0.49–0.73; p < 0.001; Table 2 and Fig 2A). There was no evidence of any interaction, and the Schoenfeld proportional hazard assumption was not violated.

### Mortality

Mortality was significantly higher in men, with increasing age, with more severe initial fracture type, with lower BMD and with other co-morbidities, such as smoking, higher alcohol intake, and corticosteroid use (Table 3). Again, given the adverse criteria to ‘trigger’ the recommendation for bisphosphonate use, these individuals had a higher underlying mortality risk. Consistent with this adverse risk profile, absolute mortality was higher in the bisphosphonate-recommended group 15.0% vs. 9.5% over a mean follow-up time of 44.9 vs. 46.4 months. However, after adjustment for the adverse risk profile (Table 3), treatment recommendation was associated with a significantly lower mortality hazard (HR: 0.79: 0.64–0.97; p = 0.021; Table 3 and Fig 2B). There was no evidence of any interaction, and the Schoenfeld proportional hazard assumption was not violated.

### Sensitivity analyses

#### Women and men analysed separately

For women, the absolute subsequent fracture risk was not significantly higher for those who were recommended bisphosphonates (n = 2100; 14.0%) compared with those who were recommended calcium and vitamin D alone (n = 1793; 12.9%; p = 0.356). However, the absolute mortality risk was significantly higher (13.9% vs. 7.5%; p < 0.001). As mentioned above, women who were recommended bisphosphonates had a significantly lower subsequent fracture risk (HR: 0.59, 95%CI: 0.47–0.74; p < 0.001). The mortality risk was lower, albeit not significantly so (HR: 0.88, 95%CI: 0.69–1.13; p = 0.333).

For men, the absolute subsequent fracture risk was not significantly higher for those who were recommended bisphosphonates (n = 434; 9.9%) compared with those who were recommended calcium and vitamin D alone (n = 684; 8.9%; p = 0.579). The absolute mortality risk was significantly higher (20.7% vs. 14.8%; p = 0.010). However, after the same adjustments as mentioned above for fracture and mortality risk factors, men who were recommended bisphosphonates had a borderline lower subsequent fracture risk (HR: 0.66, 95%CI: 0.40–1.08; p = 0.097), but mortality risk was significantly lower (HR: 0.67, 95%CI: 0.46–0.97; p = 0.035).

#### Only correctly classified patients according to pre-defined criteria

Of the 5011 patients, the recommendations for 4370 patients were according to the pre-defined criteria. Other patients were excluded from these analyses. The absolute subsequent fracture and mortality risk were significantly higher for those who were recommended bisphosphonates (n = 2416; 13.2% and 15.1%, respectively) compared with those who were recommended calcium and vitamin D alone (n = 1954; 9.0% and 7.4%, respectively; p < 0.001). After the same adjustments, those who were recommended bisphosphonates had a lower, but not significantly lower, subsequent fracture risk (HR: 0.88, 95%CI: 0.64–1.21; p = 0.432). However, the mortality risk (HR: 0.72, 95%CI: 0.52–0.98; p = 0.038) remained significantly lower.

## Discussion

Patients who were recommended oral bisphosphonates had a significantly lower adjusted mortality hazard 0.79 (0.64–0.97) and subsequent fracture risk 0.60 (0.49–0.73) compared with those recommended calcium and vitamin D alone. Amongst these fully assessed patients those, who attended the FLS and were recommended oral bisphosphonates had worse baseline characteristics with respect to age, BMD and initial fracture severity, compared to those who attended the same FLS in the same time line, but did not meet the pre-specific criteria. This was expected based on the pre-defined criteria for a recommendation for bisphosphonate treatment. Adjusting for these adverse baseline conditions, the observed mortality and subsequent fracture outcomes of the group who was recommended oral bisphosphonates were significantly lower than in the group who was recommended calcium and vitamin D alone. The survival benefit was independent of the relative fracture risk reduction. The present study suggests that it is beneficial for patients who have sustained a fracture to receive oral bisphosphonate treatment in order to reduce their risk of further fractures and, importantly, reduce premature mortality.

The sensitivity analyses generated similar findings were present in both men and women and limiting the analyses to those who meet the predefined criteria. There were no changes in the direction or estimated effect sizes but some changes in significance, presumably due to the smaller numbers in these analyses.

### Findings relative to previous studies

This finding is consistent with other population-based data,[36] one RCT [25, 37] and a meta-analysis of specific anti-osteoporosis treatments.[26] The lower mortality risk in this study (HR: 0.79) is similar to that observed in the zoledronic acid trial (HR: 0.72) and the Danish health data analysis (HR 0.73).[25, 38] Each of these studies focuses on patients who had sustained a recent fracture.

There is no clear mechanism defined for the mortality benefits in this cohort study or in the other studies.[21, 25]^,^ [38] Possible mechanisms proposed, including reduced rate of bone loss that has been shown in long term studies to be an independent predictor of mortality.[18, 39, 40] Interestingly, in the Danish study, the benefit was greater, albeit but not significantly so, in those who filled multiple prescriptions (HR 0.73 vs. 0.84).

### Limitations

At the time of this study, there were limited data on the efficacy of therapy in older individuals particularly after hip fractures. Thus these elderly infirm subjects were not referred to the FLS. These patients were expected to have a life expectancy of <6 months based on the judgment of the physician. Also, some individuals declined to attend. A relatively small number of patients was already on treatment and could therefore not be analysed as part of this comparison.

This study is an association study. Typical biases in observational studies, such as healthy user effect and immortal time bias, were avoided in this study by limiting the analysis to those people who attended the FLS clinic and were fully assessed. In this observational study, there is no formal information on uptake of and adherence to bisphosphonate therapy. It is widely understood that GPs and their patients respect the FLS and follow treatment recommendations. In any case, poor adherence would probably bias against seeing any association. On the other hand, it is possible that patients recommended bisphosphonates received other medical interventions from their GP that produced a benefit. However, since the criteria for treatment recommendations were pre-defined and analyses were not based on adherence to therapy, healthy complier and immortal time biases that confound analysis of treatment adherence per se are avoided.

### Strengths

The present study is the first that uses long-term real-world follow-up data of a large cohort across a range of post-low trauma fractures. Importantly, as distinct for typical RCT approaches in individuals with osteoporosis, this is the first study that uses the FLS approach and limits analyses to people shortly after their fracture event presentation. The only comparable study is that using zoledronic acid after hip fracture.[22, 28, 41, 42] Importantly the present analyses were limited to people who attended the Fracture Liaison Service clinic within two months of their fracture event presentation. This unique feature of all individuals being FLS assessed within a few weeks of their fracture event focuses on the time of highest risk post fracture and thus the group in whom treatment is likely to have its largest effect, as for the zoledronic acid post-hip fracture study.[22, 28, 41, 42]

Strengths of this study include the criteria for treatment recommendations were specific, pre-defined and were recorded for all individuals. Moreover, the long-term follow-up of the individual patients is an advantage as randomized clinical trials rarely have any ‘placebo’ control group extending to this duration. Another strength of this study is that it extends the mortality risk reduction findings not only to a longer time period and not only after hip fractures,[43] but also to a wider range of fragility fractures.

### Conclusion

In the context of systematic care and full assessment, these data indicate that a recommendation for specific osteoporosis treatment with oral bisphosphonates is likely to be beneficial by reducing the predicted increased risk of subsequent fracture and by improving overall survival.

While some studies have reported a reduction of mortality and subsequent fracture risk reduction related to care in before-after implementation studies,[22, 28, 41, 42] it remained to be shown whether concurrent improvements in treatment rates translate to real world improvements in health outcomes. Thus, in the present study, in which allocation to specific osteoporosis treatment recommendation (or not) was according to pre-defined criteria and follow-up during the same time period, there was a benefit in survival as well as reduction in subsequent fracture rates.

These findings indicate both fracture risk and survival benefits of pharmacotherapy for osteoporosis accrue in those at high risk soon after a prior low trauma fractures. Currently, only about one third of women and likely a smaller proportion of men are recommended specific anti-osteoporosis therapy even after a low trauma fracture.[24] The current findings indicate that treatment recommendation according to guidelines for individuals who have suffered fragility fractures has potentially benefits in terms of reduction in subsequent fracture and premature mortality.

## Supporting information

S1 Dataset Fully anonymized dataset. (XLSX)
